# Dermcidin exerts its oncogenic effects in breast cancer via modulation of ERBB signaling

**DOI:** 10.1186/s12885-015-1022-6

**Published:** 2015-02-19

**Authors:** Jasna Bancovik, Dayson F Moreira, Daniel Carrasco, Jun Yao, Dale Porter, Ricardo Moura, Anamaria Camargo, Cibely C Fontes-Oliveira, Miguel G Malpartida, Silvia Carambula, Edouard Vannier, Bryan E Strauss, Alda Wakamatsu, Venancio AF Alves, Angela F Logullo, Fernando A Soares, Kornelia Polyak, José E Belizário

**Affiliations:** 1 Department of Pharmacology, Institute of Biomedical Sciences - University of São Paulo, Av Lineu Prestes 1524, 05508-900 São Paulo, SP Brazil; 2 Jerome Lipper Multiple Myeloma Disease Center, Dana-Farber Cancer Institute - Harvard Medical School, 450 Brookline Ave. D740C, Boston, MA 02215 USA; 3 Department of Neuro-Oncology Research, Division of Cancer Medicine, University of Texas - MD Anderson Cancer Center, 1515 Holcombe Blvd, Houston, TX 77030 USA; 4 Oncology Disease Area and Developmental and Molecular Pathways Group, Novartis Institutes for Biomedical Research, 250 Massachusetts Avenue, Cambridge, MA 02139 USA; 5 Ludwig Institute for Cancer Research- Hospital Sírio-Libanês, Rua Peixoto Gomide, 316, 7th floor, 01409-000 São Paulo, SP Brazil; 6 Division of Geographic Medicine & Infectious Diseases, Tufts Medical Center, 25 Harvard Street - Tupper 729, Boston, MA 02111 USA; 7 The Cancer Institute of São Paulo, Av. Dr. Arnaldo, 251, 8th floor, 01246-000 Sao Paulo, SP Brazil; 8 Department of Pathology - School of Medicine, University of São Paulo, Avenida Dr Enéas de Carvalho Aguiar, 155 - 10th floor, 05403-000 Sao Paulo, SP Brazil; 9 Department of Pathology - Paulista School of Medicine, Federal University of São Paulo, Rua Sena Madureira, 1500, 04021-001 São Paulo, SP Brazil; 10 Department of Pathology - AC Camargo Cancer Center, Rua Professor Antônio Prudente, 211, 01509-010 São Paulo, SP Brazil; 11 Department of Medical Oncology, Dana-Farber Cancer Institute - Harvard Medical School, 450 Brookline Ave. D740C, Boston, MA 02215 USA

**Keywords:** Breast cancer, Dermcidin, ERBB signaling, Oncogene, Apoptosis

## Abstract

**Background:**

We previously identified dermicidin (DCD), which encodes a growth and survival factor, as a gene amplified and overexpressed in a subset of breast tumors. Patients with DCD-positive breast cancer have worse prognostic features. We therefore searched for specific molecular signatures in DCD-positive breast carcinomas from patients and representative cell lines.

**Methods:**

DCD expression was evaluated by qRT-PCR, immunohistochemical and immunoblot assays in normal and neoplastic tissues and cell lines. To investigate the role of DCD in breast tumorigenesis, we analyzed the consequences of its downregulation in human breast cancer cell lines using three specific shRNA lentiviral vectors. Genes up- and down-regulated by DCD were identified using Affymetrix microarray and analyzed by MetaCore Platform.

**Results:**

We identified DCD splice variant (DCD-SV) that is co-expressed with DCD in primary invasive breast carcinomas and in other tissue types and cell lines. DCD expression in breast tumors from patients with clinical follow up data correlated with high histological grade, HER2 amplification and luminal subtype. We found that loss of DCD expression led to reduced cell proliferation, resistance to apoptosis, and suppressed tumorigenesis in immunodeficient mice. Network analysis of gene expression data revealed perturbed ERBB signaling following DCD shRNA expression including changes in the expression of ERBB receptors and their ligands.

**Conclusions:**

These findings imply that DCD promotes breast tumorigenesis via modulation of ERBB signaling pathways. As ERBB signaling is also important for neural survival, HER2+ breast tumors may highjack DCD’s neural survival-promoting functions to promote tumorigenesis.

**Electronic supplementary material:**

The online version of this article (doi:10.1186/s12885-015-1022-6) contains supplementary material, which is available to authorized users.

## Background

We previously described DCD as a candidate oncogene in breast cancer based on its copy number gain and overexpression in a subset of tumors [1]. Patients with DCD-positive breast cancer are more likely to have metastatic lymph nodes, larger tumors, and worse clinical outcome [1]. We also demonstrated that overexpression of DCD enhanced cell proliferation and resistance to oxidative stress-induced apoptosis in cell culture [1]. Furthermore, we showed that DCD encodes for a secreted protein that binds to a candidate receptor present on the cell surface of breast cancer cells and neurons [1].

In normal human tissues DCD displays a restricted expression pattern with significant expression detected only in eccrine sweat glands of the skin [2] and in certain parts of the brain [1] Overexpression of DCD was reported in multiple human tumor types including melanoma, cutaneous tumors, breast, prostate, pancreatic, and hepatocellular carcinomas [1,3-9]. The 11 kDa full-length DCD protein and proteolytic peptides derived from it have been proposed to have diverse biological functions, such as acting as a growth and survival factor in breast cancer [1] and in neural cells [10,11], displaying antibacterial activity [2,12,13], and inducing cancer-associated cachexia in animal models and in cancer patients [14,15]. In addition, a recent study demonstrated that DCD may function as a proteolytic enzyme which can cleave and activate the pro-MMP-9 matrix metalloproteinase and, thus, may also promote tumor cell invasion [16].

Despite the presumed importance of DCD in tumorigenesis and neurodegenerative diseases, the molecular mechanisms behind its many physiological and pathological functions, its receptor, and the signaling pathway activated by it remain obscure. The DCD gene appears to have evolved fairly recently during evolution, as no homologous genes could be identified beyond New World Monkies based on Southern blot [1]. This apparent lack of DCD homologues in lower organisms made deciphering its biological function more difficult. Even in the human genome only two proteins show limited homology to DCD, and only one of these, lacritin (LACRT), has been characterized to some extent [17]. LACRT is closely linked to DCD at chromosome 12q13 and it is co-amplified and co-expressed with DCD in a subset of breast tumors [1]. Similar to DCD, lacritin is also a secreted survival factor and it was proposed to elicit its effects via activating a not-yet-identified G-protein coupled receptor(s) and calcium signaling [17-20]. However, it is unknown if DCD also functions via related signaling pathways.

To further investigate the function of DCD in breast cancer, here we describe the identification of a DCD splice variant (DCD-SV) and the consequences of downregulating DCD expression in the MDA-MB-361 human HER2+ breast cancer cell line and upregulating DCD in the MCF-7 human HER2- breast cancer cell line and in the SK-BR-3 human HER2+ amplified cell line. Notably, we determined that DCD might elicit its oncogenic and pro-survival effects via modulation of ERBB signaling.

## Methods

### Cell lines and tissue specimens

Breast tumor specimens were obtained from Boston area hospitals and AC Camargo Cancer Center (São Paulo, SP, Brazil). Normal human skin and placenta were collected at Hospital São Paulo (São Paulo, SP, Brazil). The use of the human specimens was approved by the institutional review boards (IRB) of the Brigham and Women’s and Massachusetts General Hospitals (Boston, MA, USA), Duke University Medical Center (Durham, NC, USA, the National Disease Research Interchange (Philadelphia, PA, USA) and AC Camargo Cancer Center (São Paulo, SP, Brazil). The need for informed consent was waived as the human specimens were deidentified. Breast cancer cell lines were previously described [1] and updated in Additional file 1: Table S1. For the generation of derivatives of the MDA-MB-361 cell line expressing DCD shRNA, we designed shRNA against different regions of the DCD transcript and subcloned them into pLKO-puro lentiviral construct. Lentivirus generation and validation of the shRNA clones was performed as previously described [21]. For generation of the MCF-7-DCD and SK-BR-3-DCD human cell lines, the full length human DCD cDNA was cloned into pcDNA3.1+ expression vector at *Bam*H1 and *Eco*R1 restriction enzyme sites. Plasmids were transfected into cells using LipofectAMINE 2000 (Invitrogen) and selected in 200–600 μg/ml G418 (Invitrogen). Transfection was confirmed by PCR and Western blot analyses as previously described [22].

### PCR, microarray, and network analyses

RNA preparation and RT-PCR analyses were conducted essentially as we described [1]. Gene expression profiling was performed by the Dana-Farber Microarray Core Facility using Affymetrix U133 Plus 2.0 chip following the manufacturer’s protocols; data was analyzed by dChip software [23]. Microrray data was deposited into GEO, accession number # GSE57578, and is available to scientific community (Additional file 2). Gene expression levels were compared pair-wise between control pLKO and each of the three DCD shRNA derivatives. Genes that displayed statistically significantly different expression in all three pair-wise comparisons were selected for further analyses using the MetaCore platform essentially as previously described [24]. Details of network analyses are included in the Supplementary Data. Quantitative RT-PCR analyses were performed using SYBR Green RT-PCR kit (Invitrogen, Carlsbad, CA) according to manufacturer’s instructions on Mx3005P® qPCR System (Agilent Technologies). REST^©^ software was used for statistical analyses [25]. Expression data is expressed as means ± SD. Primer sequences used for PCR amplifications are available from the authors upon request.

### Immunohistochemical, immunoprecipitation and immunoblotting analyses

Immunohistochemical analysis (IHC) of formalin fixed paraffin embedded cells and tissue samples was performed as previously described [1] using affinity-purified rabbit polyclonal raised against DCD synthetic peptide (RQAPKPRKQRSS) and DCD-SV synthetic peptide (RLVFGAPVNLTSIPLTSV), and commercially available antibodies to DCD as follow: G-81 mouse monoclonal [26], goat polyclonal (Santa Cruz Biotechnology, San Diego, CA) and rabbit polyclonal (Abgent Inc, San Diego, CA). The C-terminal peptides of human DCD and DCD-SV were used for target/specificity assay. Immunoblot analyses were performed as described [1]. For immunofluorescense, immunohistochemical and immunoprecipitation studies, the following mouse, human or rabbit primary and secondary antibodies were used: EGFR (sc-03), pEGFR (tyr 1173, sc 12351) (Santa Cruz Biotechnology), EGFR and ErbB-2/HER2 (pharmDX), cytokeratin-5/6 and cytokeratin-18 (DakoCytomation), Trastuzumab/Herceptin (Genentech Inc, South Francisco, CA), pMAPK 38 (tyr 180, 182), pAKT (tyr 308) (Cell Signaling Technology), α-tubulin, β-actin (Sigma-Aldrich, St. Louis, MO), and FITC-labeled goat anti-mouse or rabbit (Santa Cruz Biotechnology and Cell Signaling). To evaluate the phosphorylation status of EGFR, the MDA-MD-361 or MCF-7 cell clones were treated with recombinant EGF (Sigma) for 15 min, and cultures washed twice ice-cold PBS and lysed in immunoprecipitation buffer as described [27]. Lysates were incubated with anti-pEGFR overnight at 4°C and next with protein A- and G-Sepharose for 2 h and then the immunocomplexes were pelleted by centrifugation. Western blotting was performed as described [1].

### Cell proliferation and survival assays

For cell proliferation assays, cells were seeded at 4 × 10^3^ cells per well in 24-well plates in DMEM with 1% FCS and treated with recombinant DCD at concentrations 1 to 1000 ng/ml. Cell proliferation was determined by incubating the cells for 3–5 days in the presence of 0.1 mM bromo-2’-deoxyuridine (Oncogene Research, Cambridge, MA) followed by detection using protocols provided by the manufacturer. For cellular survival assay, 1-2 × 10^3^ cells in 96-well plates were incubated overnight and subsequently treated with different concentrations of H_2_O_2_, staurosporine, and TNF-α with cyclohexamide for 16–18 hours. Cellular viability was determined using a tetrazolium salt assay (Sigma-Aldrich, St. Louis, MO). Each experimental condition was measured in quadruplicates and each experiment was performed at least three times. Results are expressed as mean ± SEM.

### Xenograft assays in immunodeficient mice

For xenograft assays, 6-week-old female BALB/c nude mice were subcutaneously injected in the flank with 200 μl of matrigel (Becton-Dickson Biosciences, NJ) alone (control group) or mixed with 1 × 10^6^ cells from MDA-MB-361 pLKO clone (pLKO group) or MDA-MB-361 DCD shRNA clone (IBC-I group). Five animals were used in each group. Body weight, tumor mass and overall status were monitored every two days throughout 45 days. Animal weight is expressed as mean ± SD percentage of weight at injection. The mice were euthanized and organs and tumors were dissected, weighed and frozen in liquid nitrogen or fixed in 10% buffered formalin and embedded in paraffin. Xenograft experiments were repeated twice with essentially the same results. For *in vivo* therapy study, female nude mice (20–25 g) were subcutaneously injected in the dorsal flank with ~1 × 10^6^ MDA-MB-361 parenteral cells diluted 1:1 in Matrigel. When tumor volumes reached 200–300 mm^3^, mice were randomly distributed into groups in order to test the different treatment. Animals in group 1 received intraperitoneal doses of trastuzumab (20 mg/kg), animal in group 2 received a mixture of goat polyclonal anti-DCD antibodies (1 mg/Kg), named N-20, A-20 and S-19 (Santa Cruz Biotech); and animal in group 3 their combination one a week for a five weeks. Tumors were measured with a caliper every week, and volume calculated by the formula: tumor volume = (width)^2^ × length × 0.5. The body weight changes and performance status were monitored daily for 5 weeks. All animal experiments were performed according to a protocol approved by the Animal Care and Use Committee of the Institute of Biomedical Sciences, University of São Paulo.

### Statistical analyses

Results are expressed as mean ± SD. Data were analyzed by the Student’s paired t-test, one-way (or two-way) ANOVA and Fisher’s exact test as appropriate, using Prism software. For the mouse xenograft experiments, three groups of animals were compared using the exact Wilcoxon rank sum test.

## Results

### Expression of DCD and DCD-SV in normal and neoplastic tissues

**Figure 1:**
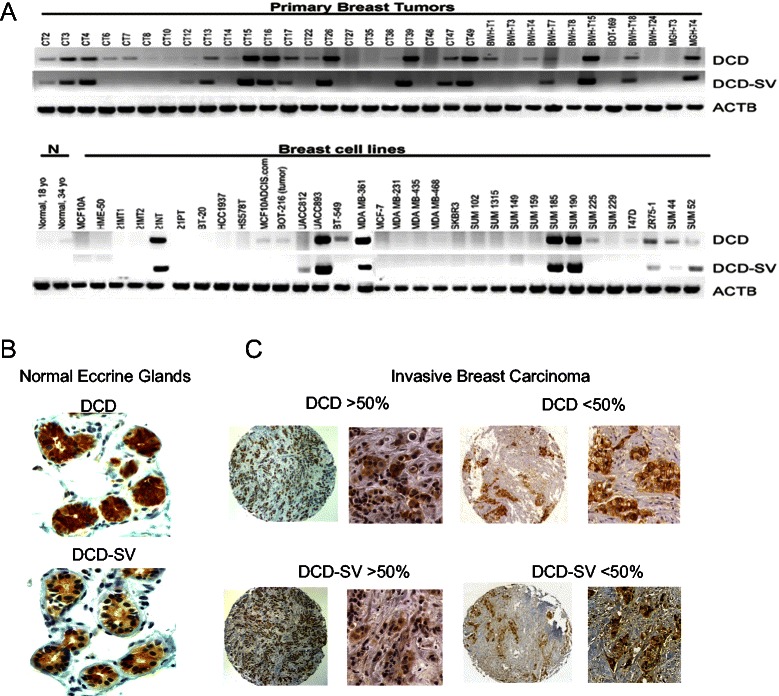
**Expression of DCD and DCD-SV in normal and neoplastic tissues. A**, RT-PCR analysis of DCD and DCD-SV expression in primary human breast carcinomas and in breast cell lines. N denotes normal breast organoids obtained from two different age women. Amplification of ACTB (actin) was used to indicate equal loading. **B**, DCD and DCD-SV immunostaining of epithelial cells and ducts of sweat gland of the skin, **C**, Representative tumor tissue sections stained with rabbit polyclonal antibodies to DCD and DCD-SV. Magnification of 40× and 200×.

While analyzing the expression of DCD by RT-PCR in various normal and neoplastic tissues and cell lines, we identified a larger transcript co-expressed with DCD. The transcript contains a different fifth exon as a result of alternative splicing (Figure 1A), thus, we designated it DCD-SV (for DCD splice variant). This 526 bp DCD-SV encodes a 12.1 kDa protein with a different C-terminus missing the hydrophobic coiled-coil structure (amino acids 80–103) thought to be essential for the antibacterial function of DCD [2]. The expression of DCD and DCD-SV correlated well in most tissue samples and cell lines analyzed, although the relative levels of the two transcripts demonstrated some variability (Figure 1A). To define relative DCD and DCD-SV expression levels more precisely, we performed quantitative RT-PCR analysis of various human tissue samples and cell lines. Among normal tissues, placenta expressed almost only DCD-SV, whereas in normal breast both transcripts were detected at a 2:1 ratio and cell lines displayed variable DCD and DCD-SV expression levels (data not shown). Another group also identified a short truncated (DCD-SV-1) and a larger (DCD-SV-2) form of DCD in human placental tissue [19]. DCD-SV-1 is expressed in villous parenchyma whereas the larger DCD-SV-2 isoform, which is similar to the DCD-SV sequence identified in our study, is expressed preferentially in reflected membrane [16].

We performed IHC using different antibodies and routinely detected the expression of DCD and DCD-SV in epithelial cells of human eccrine sweat glands (used as control) and luminal side of secretory ducts (Figure 1B). The reactivity was not present in normal mammary epithelial cells, and reliable staining was present in membrane and weaker in cytoplasm of tumor cells (Figure 1C). Next, we examined ~600 samples of primary and invasive carcinomas spotted in two tissue microarrays slides. The patient cohort was previously clinic-pathological evaluated and the tumors classified as negative or positive for estrogen and progesterone receptors and EGFR and HER2 receptors [28]. The Nottingham system was used for assessment of histologic grade of each tumor [28]. A group of 26 samples with consistent DCD immunoreactivity in <50% or >50% of tumor cells was classified into subgroups according to their clinical and pathological features. Statistically significant associations (p < 0.05) were found between DCD reactivity >50% and the subgroups with either high histological grade or with HER2 score 3 (Table 1.A). No relationship with overall survival was found. These results are in line with the findings of our previous study analyzing a smaller cohort [1].

**Table 1:** **Association of DCD expression and Breast Cancer Biomarkers**

A. Association between the clinical-pathological features and molecular markers in DCD-positive breast cancer patient samples			
*Characteristics*	Invasive Breast Carcinoma, n = 26		
*Median age, years range*	56 (37–79)		
*DCD expression*	<50%	>50%	
	no. (%)	no. (%)	*P*-value
*Nodal status*			
Negative, n = 9	7 (77.8)	2 (22.2)	0,11
Positive, n = 17	7 (41.1)	10 (58.9)	
*Histological Grade*			
Intermediate, n = 10	7 (70)	3 (30)	*0,008*
High, n = 16	2 (12.5)	14 (87.5)	
*Estrogen Receptor*			
Negative, n = 15	6 (40)	9 (60)	0,13
Positive, n = 11	8 (72.7)	3 (27.3)	
*Progesterone Receptor*			
Negative, n = 16	6 (37.5)	10 (62.5)	0,22
Positive, n = 10	7 (70)	3 (30)	
*ERBB2*			
Score 0–2, n = 14	14 (100)	0	*0,001*
Score 3, n = 12	0	12 (100)	
**B. Association between DCD and ERBBs mRNA expression in 55 breast cancer cell lines**			
	DCD expression (RMA,log2)		
	<4	≥4	
	no. (%)	no. (%)	*P*-value
*ERBB2*			
<8, n = 25	13 (23.6)	12 (21.8)	*0,047*
≥8, n = 30	7 (12.7)	23 (41.8)	
*ERBB3*			
<9, n = 21	12 (21.8)	9 (16.3)	*0,044*
≥9, n = 34	9 (16.3)	25 (45.4)	
*ERBB4*			
<4, n = 21	10 (18.1)	11 (20)	0,391
≥4, n = 34	11 f	23 (41.8)	
*EGFR*			
<7, n = 40	17 (30.9)	23 (41.8)	0,795
≥7, n = 15	5 (0.05)	10 (14.5)	

*P* = Statistical significance by Fisher’s exact.

RMA = robust multiarray average.

To further confirm the association of ERBBs and DCD expression, we compiled freely available microarray data sets of 55 human breast cancer cell lines obtained from Cancer Cell Line Encyclopedia (http://www.broadinstitute.org/ccle). The list is described in Additional file 1: Table S1 and has representative models for the different subtypes of the disease [29]. The association analyses were done across the subgroups classified as higher or lower based on whether the value was below or above the median of RMA (robust multiarray average) normalized expression value for the DCD and ERBB genes obtained in CCLE. Again, we found statistically significant association (p < 0.05) between DCD expression (RMA ≥4) with HER2 (RMA ≥8) and also with HER3 (RMA ≥9) expression (Table 1.B, Additional file 1: Table S1). As expected, in these groups are cell lines classified in the HER2 and luminal subtype, in which HER2 gene is amplified or superexpressed [29] and (Additional file 1: Table S1).

### Consequences of DCD downregulation

**Figure 2:**
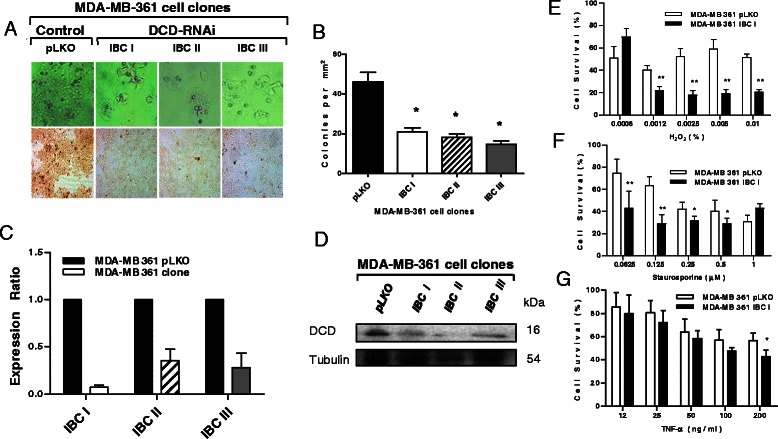
**Characterization of MDA-MB-361 cell line derivatives expressing DCD shRNA.** All experiments were repeated using three independent cell clones with essentially identical results. Representative experiments are depicted in the figures. **A**, Morphology of the cells (upper panel) and immunocytochemical analysis (lower panel) of DCD protein expression. **B**, Quantitative analysis of colony numbers (≥25 cells/colony) two weeks following plating of the cells. y-axis indicates mean colony counts ± SD; * denotes statistically significant (p < 0.05) differences. Quantitative RT-PCR **(C)** and immune-blot analysis **(D)** confirming reduced levels of DCD mRNA and protein, respectively, in DCD shRNA expressing cells. Cellular survival of control pLKO (white bars) and DCD shRNA expressing (black bars) cells following treatment with the indicated concentration of H_2_O_2_**(E)** staurosporine **(F)** and TNF-α plus cyclohexamide **(G)**. Y axis indicates % surviving cells compared to untreated controls.

To assess the function of DCD in breast cancer cells with high endogenous expression, we generated derivatives of the MDA-MB-361 human breast cancer cell line expressing three different shRNAs against DCD (IBC-I, IBC-II, and IBC-III) using pLKO plasmid-derived lentiviruses. Efficient downregulation of DCD mRNA and protein was confirmed by multiple assays including RT-PCR analyses (2C), immuno-cytochemistry (2A), and immuno-blotting (Figure 2D). Cells expressing DCD shRNAs had significantly reduced colony-forming ability (Figure 2B).

To evaluate if down-regulation of DCD affects cellular resistance to apoptosis, we exposed the cells to various doses of cytotoxic agents and found that their cellular resistance to H_2_O_2_ (Figure 2E), staurosporine (Figure 2F), and TNF-α at 200 ng/ml (Figure 2G) were significantly reduced. These results are in agreement with prior studies describing higher apoptosis resistance of cancer cells overexpressing DCD [1,6,7,9].

**Figure 3:**
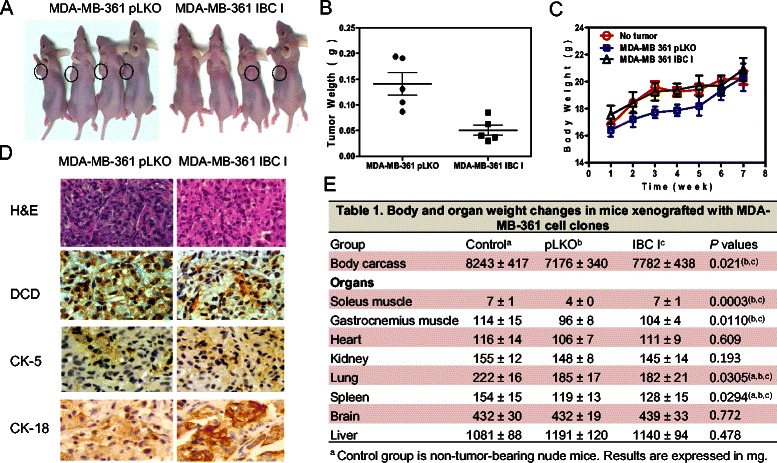
**The effect of DCD downregulation on tumorigenesis. A**, Representative mice from control and DCD shRNA experimental groups twelve weeks after injection demonstrating visible differences in tumor size. No physical signs resulting from cachexia were observed in either group of mice. **B**, Body weight of mice during the course of the experiment. Data are mean ± SE for 5–6 mice per group. *indicates statistically significant (P < 0.05) differences calculated by ANOVA. **C**, Variation of body weight along xenograft tumor growth. Data are expressed as mean ± SE of the average of weights of 5–6 animals in each experimental group. Statistical differences among groups were determined by One-way ANOVA with Turkey’s pairwise comparisons. **D**, Immunohistochemical analysis of DCD and cytokeratin 5 (CK-5) and 18 (CK-18) protein expression in xenografts derived from cells expressing DCD shRNA (IBC-I) compared to pLKO controls. **E**, Table 1 showing summary of tumor size and weight of carcass, skeletal muscles, and individual organs. Data are expressed as mean ± SE of the average of wet weights of organs of 5–6 animals in each experimental group. Statistical differences among groups were determined by One-way ANOVA with Turkey’s pairwise comparisons.

Next, we analyzed the effect of DCD downregulation on tumorigenesis by performing xenograft assays in immunodeficient mice. Mice inoculated with MDA-MB-361 cells expressing IBC-I shRNA developed smaller tumors compared to control pLKO cells (Figure 3A). The overall weight of various organs of mice inoculated with tumor cells was significantly reduced compared to animals without tumor, but no difference in body weight was observed between control and DCD shRNA expressing cells, and we did not observe cachexia in any of the experiments (Figure 3C,E). However, we observed a significant difference in tumor mass between control pLKO and IBC-I groups, which could explain the more pronounced weight losses of carcass, gastrocnemius, and soleus skeletal muscles in these mice (Figure 3E). Macroscopic local invasion or metastasis was not observed in any of the animals analyzed (data not shown). Immunohistochemical analysis confirmed decreased DCD protein levels in DCD shRNA expressing compared to control xenografts (Figure 3D). Additionally, we observed increased expression of CK-18 in DCD shRNA expressing xenografts implying more luminal phenotype that could contribute to decreased tumor growth (Figure 3D).

### Signaling pathways modulated by DCD

**Figure 4:**
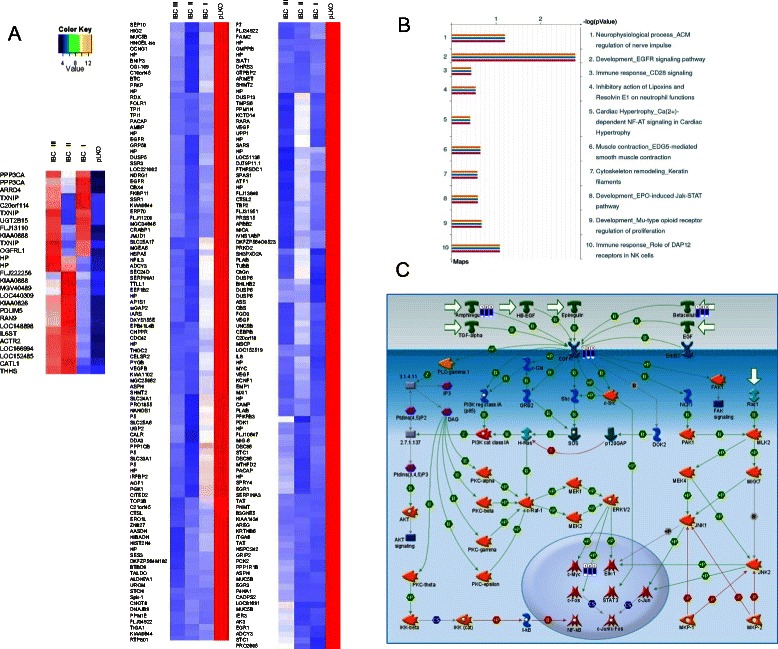
**The effect of DCD levels on global gene expression profiles. A**, Heatmap depicting relatedness of gene expression profiles of control and DCD shRNA expressing cells. Hierarchical clustering was applied to Microarray data and selected portions of the clustering heat map are shown here. Each row represents a probe and the official symbol of gene is shown. Red and green indicate high and low gene expression levels, respectively. **B**, Gene ontology biological process categories highly represented in DCD shRNA expressing cells. Categories with an enrichment score >2 using the DAVID Functional Annotation Tool are plotted. **C**, Development_ERBB signaling canonical pathway map generated by MetaCore. Blue thermometers in Betacellulin, amphiregulin, EGFR and c-Myc indicate fold-change in expression levels in each of the three different DCD shRNA expressing cell pools compared to control pLKO. See Additional file 5 for network legend.

To investigate the mechanisms underlying the growth and survival-promoting effects of DCD in MDA-MB-361 breast cancer cells, we analyzed the global gene expression profiles of control pLKO and DCD shRNA expressing cells (Figure 4A, Additional file 3: Table S3). Genes were identified as differentially expressed if their expression showed at least three-fold difference in each of the three pair-wise comparisons. Using these criteria we identified 208 up and 27 down-regulated genes (Additional file 3: Table S3). Down-regulation of DCD resulted in decreased levels of several genes that regulate oxidative stress, hypoxia, and angiogenesis including disulfide isomerase-associated 3, 4 and 6 (PDIA), stress 70 kDa protein chaperone (STCH), heat shock 70 kDa protein 5 (GRP78), hypoxia-inducible gene 2 protein (HIG2), VEGF-A, and VEGF-B. The c-MYC transcription factor, which controls the expression of numerous genes involved in metabolism, protein synthesis, and cell proliferation was also down-regulated in DCD shRNA expressing cells. Several genes regulating cell survival and death also showed altered expression in DCD shRNA expressing cells including protein phosphatase 3, the catalytic subunit of calcineurin A (PPP3CA), calcium/calmodulin-dependent protein kinase II delta (CAMK2D), thioredoxin-interacting protein (TXNIP), and cyclin-dependent kinase 6 (CDK-6). Calcineurin is a calcium and calmodulin-regulated protein phosphatase that acts as a molecular integrator of specific calcium signals. TXNIP is an inhibitor of thioredoxin, a central regulator of redox states [30]. Thus, cells overexpressing DCD may display increased resistance to oxidative stress-induced apoptosis due to their higher anti-oxidant activity potentially because thioredoxin is relieved of inhibition by TXNIP. Therefore, inhibiting DCD activity by antibodies or small molecules may increase tumor cell susceptibility to radiation and chemotherapy.

Systematic functional analysis of the differentially expressed genes using GEO revealed significant enrichment for genes with metabolic function among the 208 down-regulated genes, whereas among the 27 up-regulated genes were enriched in signal transduction pathways (Figure 4B). More detailed analysis of signaling networks and pathway using the Metacore software [24] predicted higher connectivity among the genes within the EGFR signaling canonical pathway. Betacellulin, amphiregulin, EGFR and c-Myc expression levels decreased in each of the three different DCD shRNA expressing cell pools compared to control pLKO (Figure 4C, Additional file 4: Excel Spreadsheet 1, Additional file 5).

**Figure 5:**
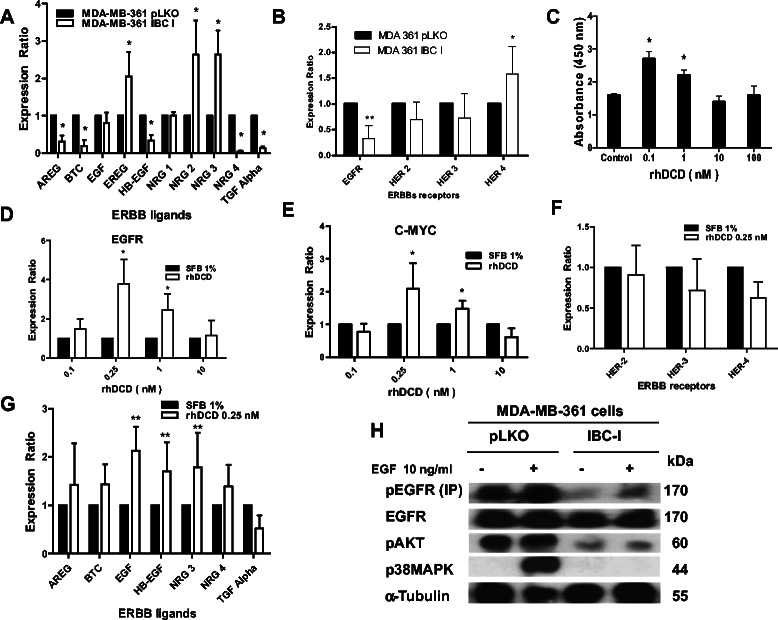
**Signaling pathways modulated by DCD. A**, Expression of ERBB family of receptors and their ligands **(B)** in control and DCD shRNA-expressing cells. Y-axis indicates mean ± SD mRNA levels (normalized to HPRT) relative to pLKO controls. **C**, Proliferation-inducing activity of highly purified recombinant human DCD in MDA-MB-361 based on BrdU incorporation assay. Columns indicate mean of absorbance units, bars, SD. Real time PCR analyses of EGFR **(D)**, C-myc **(E)**, ERBB ligands **(F)** and ERBB receptors **(G)** at the indicated doses of rhDCD. Each sample was analyzed in three independent experiments performed in duplicates. * and ** denotes P < 0.05 and P <0.01, respectively, by Student’s *t* test. **H**, effects of EGF treatment on the phoshorylation of EGFR and downstream signaling proteins p38-MAPK and pAkt in MDA-MB-361 control and DCD shRNA-expressing following of treatment with EGF 10 ng/ml for 15 minutes.

To experimentally validate these predictions of network analysis studies, we analyzed the expression levels of all ERBB family members by real time PCR in control and DCD shRNA expressing cells (Figure 5A,B). These analyses indicated that the expression levels for EGFR, ERBB2, and ERBB3, and their ligands BTC, EGF, TGF-α, AREG, HB-EGF, NGR1, and NGR4 were down-regulated, whereas the ERBB4 and ligands EREG and NGR2 were up-regulated in cells expressing DCD shRNAs. The reduction of EGFR protein levels was confirmed by immunohistochemical analysis of xenografts derived from DCD shRNA expressing cells (Additional file 6). To analyze signaling changes downstream of ERBB receptors, we analyzed the phosphorylation status of EGFR, Akt and p42/44 MAPK in control and DCD shRNA expressing MDA-MB-361 cells untreated or treated with EGF 10 ng/ml. The phosphorylation of EGFR, Akt, and MAPK proteins increased in control pLKO cells but was low or no detectable in IBC-I cells (Figure 5H).

Interestingly, ERBB4 promotes differentiation in mammary epithelial cells [31,32] and it is associated with better prognosis in breast cancer patients [33,34]. ERBB4 may execute its differentiation-inducing function by dimerizing with other ERBB family members and decreasing the levels of more oncogenic ERBB heterodimers such as ERBB3/ERBB2 [35]. Correlating with this, MDA-MB-361 breast cancer cells expressing DCD shRNA displayed a more differentiated luminal epithelial cell phenotype compared to control cells (Figure 2A). The combined regulation of c-MYC, ERBBs, and several other signal pathways by DCD may play a role in this process. Thus, an intriguing hypothesis based on our data is that the physiologic function of DCD is to promote progenitor-like cellular phenotype via modulating the activity of pathways involved in maintaining stem cell states.

### Consequences of DCD overexpression or treatment

**Figure 6:**
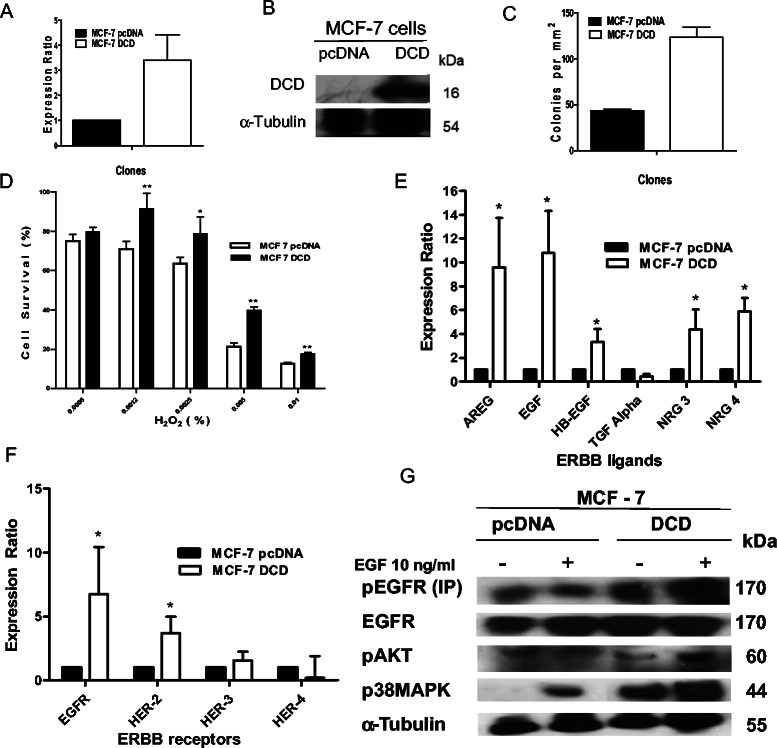
**Characterization of MCF-7 cells stably expressing DCD.** Quantitative RT-PCR **(A)** and western blot **(B)** analysis confirming the expression of DCD mRNA and protein in MCF-7-DCD cells. DCD transcript levels were normalized to that of HPRT using REST software and the expression level in MCF-7 pcDNA control was designated as 1. **C**, Quantitative analysis of colony numbers (>25 cells/colony) two weeks after plating of the cells. **D**, Cellular survival of control and DCD overexpressing MCF-7 cells treated with H_2_O_2_, at indicated concentration, for 16 hrs. Cell survival rates were estimated by MTT assay. Y-axis indicates % surviving cells as percentage of untreated control. Results are given as means ± SD of three experiments. * p < 0,01 and ** p < 0,001 by Student t test. Quantitative RT-PCR analyses showing the fold-increases in the mRNA expression of ligands **(E)** and the receptors **(F)** of ERBB family in DCD expressing MCF-7 cells compared to control. Y-axis indicates mean ± SD mRNA levels (normalized to HPRT) relative to controls. **G**, Effects of EGF treatment on the phosphorylation of EGFR and downstream signaling proteins p38-MAPK and pAkt in MCF-7 control and DCD-expressing cells following the treatment with EGF 10 ng/ml for 15 min.

To further explore the relationship between DCD expression and ERBB signaling pathways, we generated derivatives of the MCF-7 estrogen receptor positive and HER2-non-amplified luminal breast cancer cells stably expressing DCD. We compared cell growth and survival in control and DCD-expressing cells as well as the expression levels of ERBB family of receptors and ligands and components of their signaling pathways (Figure 6). DCD overexpression increased colony formation and survival (Figure 6C,D) as well as xenograft growth in immunodeficient mice (data not shown). Similar observation was described previously [36]. The constitutive autocrine expression of DCD significantly increased the mRNA levels of EGFR, HER2/ErbB2, AREG, EGF, HB-EGF, NRG3, and NRG4 (Figure 6E,F). Following EGF stimulation, DCD-expressing MCF-7 cells displayed more pronounced phosphorylation of EGFR, Akt, and MAPK proteins compared to MCF-7 pCDNA cells (Figure 6G). Next, we demonstrated that the overexpression of DCD gene in SK-BR-3 cells increase the proliferation of this HER2-amplified cell line as well as tumor growth when the cells were implanted in the mammary fat pads of female immunodeficient mice (Additional file 7: Figure S3). Thus, the pattern of changes observed in these two DCD overexpressing cells are the opposite of those found in MDA-MB-361 cells expressing DCD shRNAs strengthening the link between DCD and ERBB signaling.

**Figure 7:**
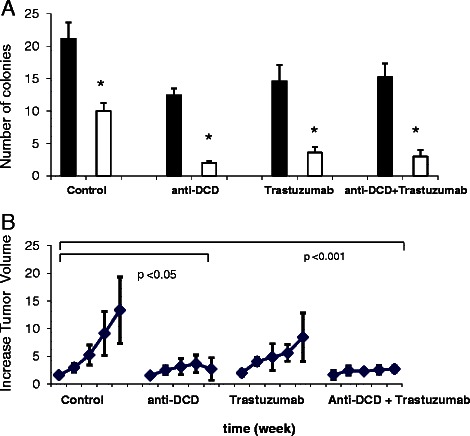
**Enhanced effect of anti-DCD rabbit polyclonal antibodies and trastuzumab combination on growth and tumor formation of MDA-MB-361 cells*in vitro*and*in vivo*. A**, Quantitative analysis of colony numbers observed 72 hours after inoculation of MDA-MB-361 cells in 96-well plates in the presence of 10% BFS (black column 1) or in the presence of 1% BFS plus non-specific antibodies (white column 1), or 1% BFS in the presence of non-specific antibody (black column 2, 3 and 4) or presence of the antibodies trastuzumab (20 mg/kg), a mixture of goat polyclonal anti-DCD antibodies or their mixture at final concentration of 100 μg/ml. Colonies numbers with ≥5 cells/colony were counted manually in at least 10 fields. Results are given as means ± SD of three experiments and statistical difference (p < 0,05) were determined by Student *t* test. **B**, Fold-increase in the volume of MDA-MB-361 xenograft tumors growing in mice (4–5 animal in each group) untreated or treated with weekly i.p. doses of trastuzumab (20 mg/kg), a mixture of goat polyclonal anti-DCD antibodies (1 mg/kg), named N-20, A-20 and S-19 or their combination for a five weeks. Statistical difference (p < 0,05) were determined by One-way ANOVA with Turkey’s pairwise comparisons.

To demonstrate that the observed effects were due to the extracellular actions of DCD, we analyzed the proliferation of MDA-MB-361 cells treated with 1–100 nM of highly purified recombinant human DCD (rhDCD) (Figure 5C). Similar to our prior findings [1], recombinant DCD enhanced cellular proliferation at 0.1-1 nM but not at 10 and 100 nM. Similar bell-shaped dose–response curves have been observed in experiments that established Y-P30, a DCD-derived peptide, as a neural survival peptide [1,10,11], lacritin, a homolog to DCD [19] and other well-known mitogenic factors including sonic hedgehog, VEGF, FGF, and PDGF. More importantly, real time RT-PCR analyses of treated cells confirmed the upregulation of EGFR, c-MYC, EGF, HB-EGF, and NRG3 (Figure 5D,E,F), whereas the expression of HER-2, −3 and −4 receptors and ligands AREG, BTC, TGF-α, and NRG4 did not change significantly (Figure 5F,G). Finally, we tested the efficacy of trastuzumab, a humanized polyclonal antibody against HER2, and goat polyclonal antibodies against DCD for the treatment of parental MDA-MB-361 cells *in vitro* and *in vivo* (Figure 7A,B). The results demonstrated that individually or the combination of anti-DCD antibodies and trastuzumab caused a significant reduction of the number of cell colonies in cell culture as well as the tumor growth as xenograft in immunodeficient mice. These results confirm our hypothesis that DCD autocrinally produced by MDA-MB-361 cells may be acting in concert with the ligands of HER/ErbB receptor family to stimulate the growth and proliferation of breast cancer cells.

## Discussion

Here, we describe for the first time the co-expression of DCD and DCD-SV in normal skin tissue and breast cancer cell lines using validated and novel specific antibodies against different portions of these proteins. The DCD splice variant is identical in its nucleotide sequence to a larger (DCD-SV-2) form of DCD identified in human placental tissue as described [37]. Although we found an association of DCD with either high histological grade or with HER2 positive samples (score 3), we did not find a significant relationship between tumor samples having DCD reactivity and overall survival in this small cohort of 26 breast cancer patients. These results are in line with our previous studies [1,22,38].

There are few studies published in the literature that report possible molecular mechanism(s) by which DCD native protein and DCD-generated peptides exert their function as growth and survival factors and antibiotic peptides [6-13]. We believe that the binding to low and high affinity membrane receptors [1] would directly or indirectly promote the integration of network signaling pathways leading ultimately to EGFR phosphorylation and activation of p38 MAPK and Akt (Figures 5 and 6). A recent study has described the crystal and atomic structure of DCD-1 L antibiotic peptide and detailed a mechanism by which individual peptides undergo oligomerization and assembly into a channel structure with ion conductivity properties across a biomimetic membrane [37]. It is not known if this putative channel is formed in mammalian membranes nor if it influences the growth rate of malignant cells.

Human breast cancer cells selected for resistance to trastuzumab *in vivo* overexpress epidermal growth factor receptor and ErbB ligands and remain dependent on the ErbB receptor network [27,35,39]. Our experiments in Figure 7 provide further evidence for parallel pathways and their possible mediators, for example, DCD. Finally, it is important to mention a recent study published by Wilhelm and colleagues [40] confirming the biological role of DCD as biomarker for cellular resistance of various tumor cells to the EGFR/ErbB1 tyrosine kinase inhibitors erlotinib and lapatinib.

## Conclusions

Using gain-of-function and loss-of-function approaches we confirmed that DCD acts as a growth and survival-promoting factor in breast cancer. Furthermore, we demonstrated that these effects are due to the modulation of ERBB receptor signaling by DCD. In agreement with this, here and in a previous study [1], we found that DCD-expressing breast tumors are frequently HER2+. Our data also imply that DCD-expressing HER2+ tumors may be more likely to be resistant to HER2-targeted therapies; a hypothesis that worth investigating in future studies.

## Additional files

Additional file 1: Table S1. ER/PR/ERBBs/DCD status, source, clinical and pathological features of the breast cancer cell lines. **Figure S1.** The average of RMA (robust multiarray average) normalized expression values for DCD, HER2, HER3, HER4 and EGFR in 55 breast cancer cell lines.
Additional file 2:
**GEO deposit GSE57578 of microarray data.**

Additional file 3: Table S2. Microarray gene expression data for MDA-MB-361 control pLKO and DCD shRNA expressing subclones.
Additional file 4: Table S3. Bioinformatic analysis of signaling networks and pathways using MetaCore Software.
Additional file 5: Table S4. Legend to symbols and objects on Figure 4B.
Additional file 6: Figure S2. Representative immunohistochemical (IHC) analysis of EGFR, HER-2/ErbB-2 and HER-4/ErbB4 in xenografts derived from control and DCD shRNA expressing MDA-MB-361 cells.
Additional file 7: Figure S3. Characterization of SK-BR-3 stably expressing DCD gene and tumor growth as xenograft in immunodeficient mice.

## Abbreviations

EGF: Epidermal growth factor receptor
EGFR: Epidermal growth factor receptor
HER2: Human epidermal growth factor receptor 2
HER3: Human epidermal growth factor receptor 3
RT-PCR: Reverse transcriptase polymerase chain reaction
qPCR: Quantitative PCR
pMAPK 38: Phosphorylated p38 mitogen-activated protein kinase 38
DMEM: Dulbecco’s modified Eagle’s medium
FCS: Fetal calf serum
TNF-α: Tumor necrosis factor-α
SEM: The standard error of the mean
SD: The standard deviation
ANOVA: Analysis of variance
shRNA: Short hairpin RNA
VEGF: Vascular endothelial growth factor
BTC: Betacellulin
TGF-α: Transforming growth factor-α
AREG: Amphiregulin
HB-EGF: Heparin-binding EGF-like growth factor
NGR: Neuregulin
GDF-15: Growth differentiation factor 15
Akt: Protein Kinase B (PKB)
