# Supplementary material for: Dermcidin exerts its oncogenic effects in breast cancer via modulation of ERBB signaling
Source: BMC Cancer. 2015 Feb 19;15:70. doi: 10.1186/s12885-015-1022-6 (PMC4353460; doi:10.1186/s12885-015-1022-6)
Supplement: Additional file 1: Table S1. — ER/PR/ERBBs/DCD status, source, clinical and pathological features of the breast cancer cell lines. Figure S1. The average of RMA (robust multiarray average) normalized expression values for DCD, HER2, HER3, HER4 and EGFR in 55 breast cancer cell lines. [file 12885_2015_1022_MOESM1_ESM.pdf]

Table S1. ER/PR/ERBBs/DCD status, source, clinical and pathological features of the breast cancer cell lines

Cell Lines	ER	PR	DCD </≥4	HER2 </≥8	HER3 </≥9	HER4 </≥4	EGFR </≥7	Source	Tumor Type	Subtype
EFM-192A	pos		<	>,A	>	<	<	PE	IDC	Lu
EVSA-T	neg	pos	>	>	>	>	<	PE	IDC	Lu
MDA-MB4-53	neg	neg	>	>,A	>	>	>	PF	AC	Lu
MDA-MB-231	neg	neg	>	<	<	>	>	PE	AC	B
BT-549	neg	neg	>	>	<	<	>	P.Br	IDC	B
CAL85-1	neg	neg	>	>	>	<	>	PE	IDC	B
EFM-19	pos	pos	>	>	>	<	<	PE	Duc.Ca	Lu
BT-474	pos	pos	<	>,A	>	>	<	P.Br	IDC	Lu
Hs.606T			<	<	<	<	<		N	
MDA-MB-415	pos	neg	>	<	>	>	>	PE	AC	Lu
AU565	neg	neg	>	>,A	>	>	>	PE	AC	Lu
Hs.578T	neg	neg	>	<	<	>	>	P.Br	AC	B
BT-20	neg	neg	>	>	>	<	>	P.Br	IDC	B
MDA-MB-361	pos	neg	<	>,A	<	<	>	P.Br	AC	Lu
MDA-MB-436	neg	neg	<	<	<	<	<	PE	IDC	B
SK-BR-3	neg	neg	>	>,A	>	<	>	PE	AC	Lu
HCC1143	neg	neg	<	<	<	>	>	P.Br	IDC	B
HCC1937	neg	neg	<	<	<	<	>	P.Br	Duc.Ca	B
HCC1569	neg	neg	>	>,A	>	>	<	P.Br	MBC	B
HCC1395	neg	neg	<	<	<	>	<	P.Br	Duc.Ca	B
HCC2218	neg	neg	>	>,A	>	<	<	PE	IDC	Lu
HCC1954	neg	neg	<	>,A	<	>	<	P.Br	Duc.Ca	B
CAMA-1	pos	neg	>	>	>	>	<	PE	AC	Lu
HMC-1-8			<	<	<	>	<	PE	IDC	
HCC2157	neg	neg	>	>	>	>	<	P.Br	Duc.Ca	B
Hs.739T			<	<	<	<	<	P.Br	AC	
HCC38	neg	neg	>	>	>	<	<	P.Br	Duc.Ca	Lu
DU-4475	neg	neg	>	<	<	<	<	P.Br	Duc.Ca	B
MDA-MB-175VI	pos	neg	>	>,A	>	<	<	PE	IDC	Lu
YMB-1	pos	neg	>	>	>	>	<	AF	IDC	

T47-D	pos	pos	>	>	>	>	<	PE	IDC	Lu
ZR-75-1	pos	neg	>	>	>	<	<	AF	IDC	Lu
HCC70	neg	neg	<	>	>	>	<	P.Br	Duc.Ca	B
MCF-7	pos	pos	>	>	>	>	<	PE	IDC	Lu
HCC202	neg	neg	>	>,A	>	<	<	P.Br	Duc.Ca	Lu
MDA-MB-157	neg	neg	<	<	<	>	<	PE	MC	B
HCC1419	neg	neg	<	<	>	>	<	PE	AC	Lu
BT-483	pos	pos	>	>	>	>	<	P.Br	IDC	Lu
Hs.281.T			>	<	<	<	<	P.Br	AC	
HCC1428	pos	pos	>	<	>	>	<	PE	AC	Lu
ZR-75-30	pos	neg	>	>,A	>	>	<	AF	IDC	Lu
Hs.343T			>	<	<	<	<	AC	Duc.CA	
CAL-51	neg	neg	<	<	<	>	<	PE	AC	B
CAL-120	neg	neg	>	<	<	>	<	PE	IDC	B
JIMT1	neg	neg	>	>A	>	<	>	PE	IDC	Lu
HCC1806	neg	neg	>	<	>	>	>	P.Br	ASC	B
UACC-812	pos	neg	<	>,A	>	<	<	P.Br	IDC	Lu
CAL-148	neg	neg	>	>	>	>	<	PE	IDC	Lu
MDA-MB-134VI	pos	neg	>	<	>	>	>	PE	IDC	Lu
HCC1500	pos	pos	<	<	>	>	<	P.Br	Duc.Ca	B
HCC1599	neg	neg	>	<	>	>	<	PE	Duc.Ca	B
UACC-893	neg	neg	>	>,A	>	>	>	P.Br	Duc.Ca	Lu
HDQ-P1	neg	neg	<	<	>	<	<	PE	IDC	B
Hs.742T			>	<	<	<	<	P.Br	AC	
KPL-1	pos	neg	<	<	>	>	<	PE	Duc.Ca	Lu
Other cell lines used in this study										
21MT1	neg	pos	<	>,A				PE	Lung Met	
21MT2	neg	pos	<	>,A				PE	Lung Met	
21NT	neg	pos	>	>,A				P.Br	Duc.Ca	
21PT	neg	pos	<	>,A				P.Br	Duc.Ca	
HME50	neg	neg	<	<				P.Br	N	B
MCF-10A	neg	neg	<	<				P.Br	N	B
SUM-102PT	neg	neg	<	<	<	<	<	P.Br	AC	B
SUM-1315	neg	neg	<	<			>	P.Br	Duc.Ca	B

SUM-149PT	neg	neg	<	<	<	<	>	PE	Inf.Duc.Ca	B
SUM-159PT	neg	neg	<	<			>	PE	Ana.Ca	B
SUM-185PE	neg	neg	>	<	<			PE	Ana.Ca	Lu
SUM-190PT	neg	neg	>	>,A	>	<	>,A	PE	Inf.Duc.Ca	Lu
SUM-225CWN	neg	neg	<	>,A	>	<	>	PE	Inf.Duc.Ca	B
SUM-229PE	neg	neg	<	<			>	PE	IDC	B
SUM-44	pos	neg	>	<			>,A	PE	Ca	Lu
SUM-52	pos	neg	>	<			>,A	PE	Ca	Lu

The ER/PR/HER2 status and subtypes were obtained from Neve RM et al. 2006 (35), ATCC (http://www.atcc.org) and Asterand (http://www.asterand.com). The gene expression (GE) profile in 55 breast cancer cell lines used in this study is available at the Cancer Cell Line Encyclopedia (http://www.broadinstitute.org/ccle). The Affymetrix probes 117159_at, 2064_at and 2065_at, 2066_at and 1956_at were chosen to obtain mRNA expression for DCD, HER2, HER3, HER4 and EGFR, respectively. The median of RMA (robust multiarray average) normalized expression value, log2, for each gene (DCD=4; HER2=8; HER3=9; HER4=4 and EGFR=7, respectively) was used to classify subgroups of cell line as lower (indicated as <) or higher expression (indicated as ≥) based on whether the value was below or equal or above the median for each gene across the 55 breast cancer cell lines. The relative level of DCD mRNA was confirmed in the majority of cell line using PCR assays as reported here (Figure 1) and elsewhere (Porter et al., 2003). Previous studies have analyzed the ER, PR and HER2 mRNA expression and confirmed the levels of expression correlates with those obtained using IHC and FISH assays (Neve RM et al. 2006).

Abbreviations: ER, estrogen receptor; PR, progesterone receptor; pos, positive; neg, negative; P.Br, primary breast; PE, pleural effusion; AF, ascites fluid; AC, adenocarcinoma; DuCa, ductal carcinoma; IDC, invasive ductal carcinoma; F, fibrocystic disease; Inf.BrCa, inflammatory carcinoma; Ana.Ca, anaplastic carcinoma; LungMeta, lung metastasis; MC, metaplastic carcionoma; ASC, acantholytic squamous carcinoma; Ca, carcinoma; B, basal; Lu; luminal; W, white; B, black; EL, East Indian; >,A, higher-amplified; N, normal.

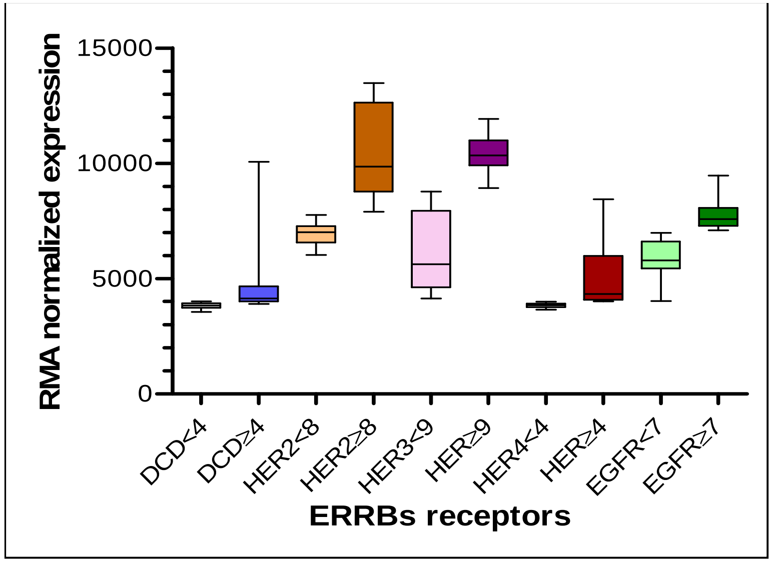

Figure S1. The average of RMA (robust multiarray average) normalized expression values for DCD, HER2, HER3, HER4 and EGFR in 55 breast cancer cell lines (Table S1). The subgroups of cell lines were classified as lower (indicated as <) or higher expression (indicated as >) based on whether the value was below or equal or above the median for each gene across the 55 breast cancer cell lines. The groups were compared by unpaired *t* test and the differences between groups were statistically significant ($p<0.005$).
