# Supplementary material for: Dermcidin exerts its oncogenic effects in breast cancer via modulation of ERBB signaling
Source: BMC Cancer. 2015 Feb 19;15:70. doi: 10.1186/s12885-015-1022-6 (PMC4353460; doi:10.1186/s12885-015-1022-6)
Supplement: Additional file 2: — GEO deposit GSE57578 of microarray data. [file 12885_2015_1022_MOESM2_ESM.pdf]

Series GSE57578	Query DataSets for GSE57578
Status	Public on May 13, 2014
Title	down-regulation of dermcidin by shRNA in breast cancer cell line MDA-MB-361
Organism	Homo sapiens
Experiment type	Expression profiling by array
Summary	DCD is a gene amplified and overexpressed in a subset of breast tumors acting as a growth and survival factor. Patients with DCD-positive breast cancer have worse prognostic features. To investigate the role of DCD in breast tumorigenesis, we analyzed the consequences of its downregulation in human breast cancer cell lines using three specific shRNA lentivirus vectors. Genes up- and down-regulated by DCD were identified using Affymetrix microarray and analyzed by MetaCore Platform. We found that loss of DCD expression led to reduced cell proliferation, resistance to apoptosis, and suppressed tumorigenesis in immunodeficient mice. Network analysis of gene expression data revealed perturbed ERBB signaling following DCD shRNA expression including changes in the expression of ERBB receptors and their ligands. These findings imply that DCD promotes breast tumorigenesis via modulating the activity of the ERBB signaling pathways. As ERBB signaling is also important for neural survival, HER2+ breast tumors may highjack DCD's neural survival-promoting functions to promote tumorigenesis.
Overall design	MDA-MB-361 human breast cancer cell line expressing DCD-targeting shRNA in pLKO vector (clones 1, 2 and 3) and pLKO control vector
Contributor(s)	Markovic J, Moreira DF, Carrasco D, Yao J, Porter D, Moura R, Camargo A, Fontes-Oliveira CC, Malpartida MG, Carambula S, Vannier E, Strauss BE, Wakamatsu A, Alves VA, Logullo AF, Soares FA, Polyak K, Belizario JE
