# Supplementary material for: Dermcidin exerts its oncogenic effects in breast cancer via modulation of ERBB signaling
Source: BMC Cancer. 2015 Feb 19;15:70. doi: 10.1186/s12885-015-1022-6 (PMC4353460; doi:10.1186/s12885-015-1022-6)

Network Objects

Click on any object in the network to obtain class info

Enzymes

Generic enzyme

KINASE

Generic kinase

Protein kinase

Lipid kinase

PHOSPHATASE

Generic phosphatase

Protein phosphatase

Lipid phosphatase

PHOSPHOLIPASE

Generic phospholipase

PROTEASE

Generic protease

Metalloprotease

GTPase

G-alpha

RAS - superfamily

Channels/Transporters

Generic channel

Ligand-gated ion channel

Voltage-gated ion channel

Transporter

Receptors

Generic receptor

GPCR

Receptors with enzyme activity

Generic classes

Receptor ligand

Transcription factor

Protein

Cell membrane glycoprotein

Compound

Predicted metabolite or user's structure

Inorganic ion

Reaction

DNA

RNA

Generic binding protein

G protein adaptor/regulators

G beta/gamma

Regulators (GDI, GAP, GEF)

Groups of objects

A complex or a group

Proteins or compounds physically connected into a complex or related as a group

Logical association

Proteins or compounds linked by logical relations. Associations may be closed or opened up by click on object

Custom association

Group 1

Group of collapsed objects chosen by user

Object highlighting

Nodes and root nodes

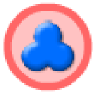

Found object

Object selected on the search pane

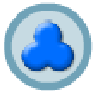

Manually selected node(s)

Object(s) selected by ctrl + click on it or by click + drag rectangle around it

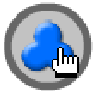

Highlight by mouse over

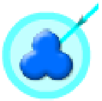

Highlight upstream objects

When the mouse is over an object (node on a network), the closest interacting nodes are highlighted in CYAN if the direction of interaction is **from** the initial object

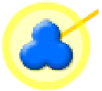

Highlight downstream objects

When the mouse is over an object (node on a network), the closest interacting nodes are highlighted in yellow if the direction of interaction is **towards** the initial object

Root nodes

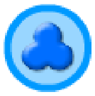

Root node(s) for network expansion (building)

Object(s) from a user-specified uploaded list or from experiments

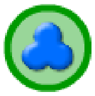

Initial object(s)

Object(s) chosen to build the pathways **from**

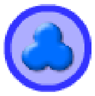

Intermediate object(s)

Object(s) situated along the pathway

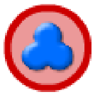

Terminal object(s)

Object(s) the pathways terminate **on**

Possible combinations of three above marks (except the first one)

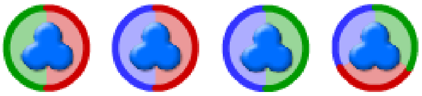

Expression data

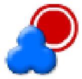

Overexpressed gene(s)

Genes with higher conditional expression level compared to the experimental "control"

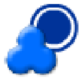

Underexpressed gene(s)

Genes with lower conditional expression level compare to the experimental "control"

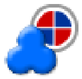

Mixed-expressed gene(s)

Genes with conditional expression level statistically different from the experimental "control", with the "sign" of expression varying in different experiments

Other marks

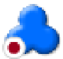

Red circle

The links terminated due to a restriction of the number of steps in network expansion.
Network may be expanded from such nodes

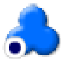

Blue circle

The links terminated due to network truncation.
Network may be expanded from such nodes

Interactions between objects

Click on any hexagon in the networks for interaction annotation

Link legend

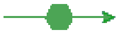

Positive effect

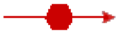

Negative effect

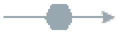

Unspecified effect

Technical link

Mechanisms

Physical interactions

B

Binding

Protein or compound binds other protein or compound

C

Cleavage

Cleavage of a protein at a specific site yielding distinctive peptide fragments.
Proteolytic cleavage can be carried out by both enzymes and compounds

CM

Covalent modifications

(neddylation/deneddylation, sumoylation/desumoylation, ubiquitination/deubiquitination and etc.) Protein activity regulation by covalent binding of a small chemical group to the aminoacids of an active site.

+P

Phosphorylation

Protein activity regulation by an addition of a phosphate group

-P

Dephosphorylation

Protein activity regulation by a removal of a phosphate group

T **Transformation**

Protein activity regulation by binding & hydrolysis of GTP

Tn **Transport**

Transport of a protein or a compound between organelles

Z **Catalysis**

Catalysis of an enzymatic reaction

Tr **Transcription regulation**

Physical binding of a transcription factor to target gene’s promoter

M **MicroRNA binding**

Regulation of gene expression by binding of microRNA to target mRNA

Functional interactions

IE **Influence on expression**

Protein’s or compound’s action results in changing the expression level of target gene(s)

Cn **Competition**

Protein activity regulation by competition at the substrate binding site

? **Unspecified interactions**

Mechanism is unknown or/and effect is indirect

PE **Drug-Drug interactions. Pharmacological effect**

Drugs change pharmacological effects of other drugs, for instance by competing for drug metabolism enzymes or organic transporters

TE **Drug-Drug interactions. Toxic effect**

Drugs change toxic effects of other drugs, for instance by competing for drug metabolism enzymes or organic transporters

Logical relations

GR **Group relation**

Object belongs to a generic group of related objects

CS **Complex subunit**

Protein is a subunit of a protein complex

SR **Similarity relation**

Chemically similar compounds with chosen Tanimoto similarity score

Connectors

Connectors

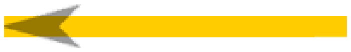

Incoming interaction

When the mouse is over an object, yellow link indicates direction **to** the object

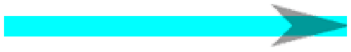

Outgoing interaction

Cyan link indicates direction **from** the object

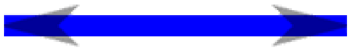

Bidirectional interaction

Blue link indicates **BI-DIRECTIONAL** interaction

Non-directional link

Blue link also indicates an interaction for which the direction is not specified

Traced link

The link is always highlighted in blue if both linked objects are selected in "Trace" mode

Interactions from custom list

Interaction is in the network

Interaction is represented by a thin solid line and is highlighted in blue

Interaction is in the base, but not in network

Interaction is highlighted in yellow

Interaction is not present in the base

Interaction is highlighted in magenta

Canonical pathways

Canonical pathways

The link is highlighted in thick cyan line

Custom marked links (user's choice)

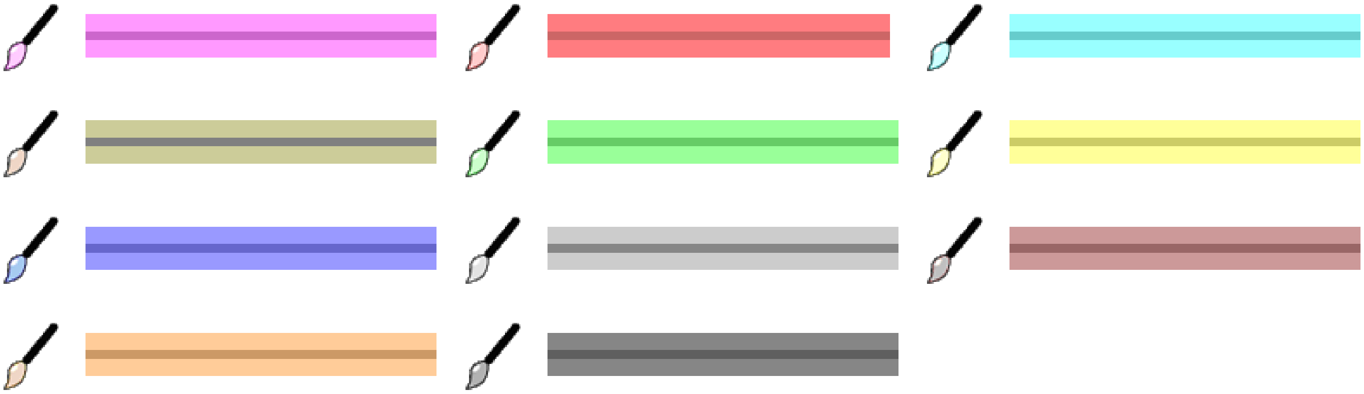

Supplement: Additional file 5: Table S4. — Legend to symbols and objects on Figure 4B. [file 12885_2015_1022_MOESM5_ESM.pdf]
