# Supplementary material for: Dermcidin exerts its oncogenic effects in breast cancer via modulation of ERBB signaling
Source: BMC Cancer. 2015 Feb 19;15:70. doi: 10.1186/s12885-015-1022-6 (PMC4353460; doi:10.1186/s12885-015-1022-6)
Supplement: Additional file 6: Figure S2. — Representative immunohistochemical (IHC) analysis of EGFR, HER-2/ErbB-2 and HER-4/ErbB4 in xenografts derived from control and DCD shRNA expressing MDA-MB-361 cells. [file 12885_2015_1022_MOESM6_ESM.ppt]

## Slide 1
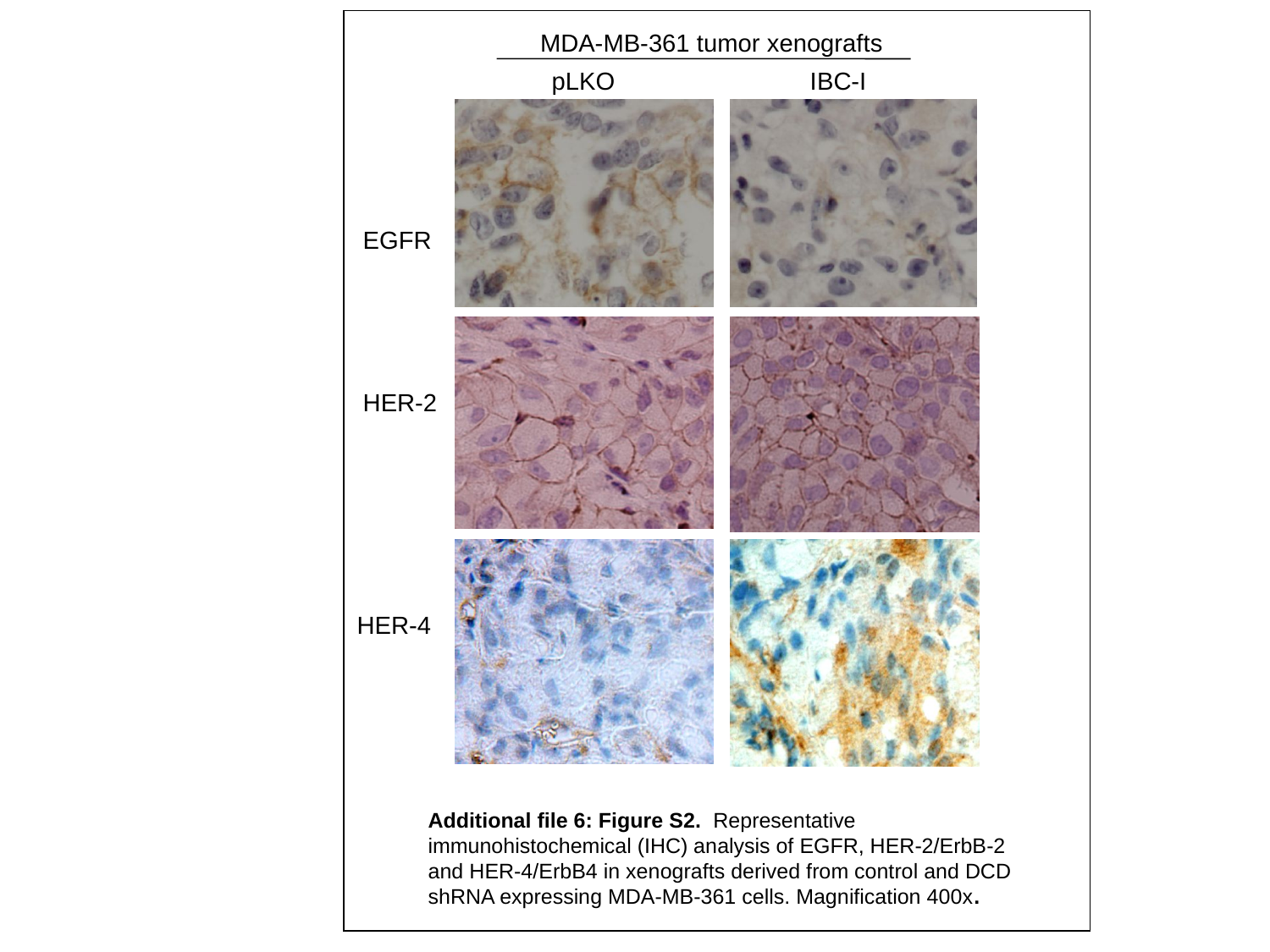

MDA-MB-361 tumor xenografts
pLKO IBC-I
EGFR
HER-2
HER-4
Additional file 6: Figure S2. Representative immunohistochemical (IHC) analysis of EGFR, HER-2/ErbB-2 and HER-4/ErbB4 in xenografts derived from control and DCD shRNA expressing MDA-MB-361 cells. Magnification 400x.
