# Supplementary material for: Dermcidin exerts its oncogenic effects in breast cancer via modulation of ERBB signaling
Source: BMC Cancer. 2015 Feb 19;15:70. doi: 10.1186/s12885-015-1022-6 (PMC4353460; doi:10.1186/s12885-015-1022-6)
Supplement: Additional file 7: Figure S3. — Characterization of SK-BR-3 stably expressing DCD gene and tumor growth as xenograft in immunodeficient mice. [file 12885_2015_1022_MOESM7_ESM.pdf]

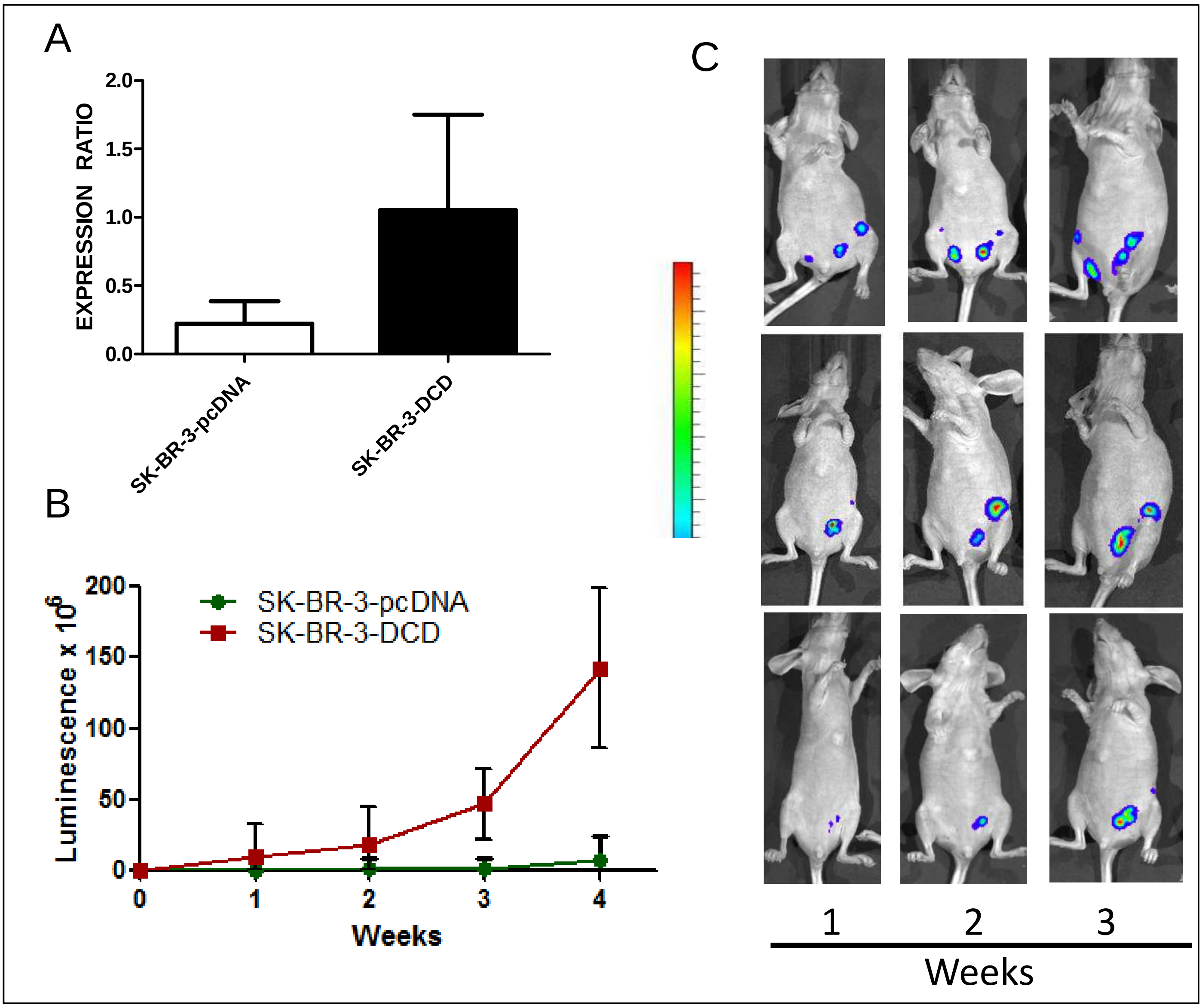

Figure S6. Characterization of SK-BR-3 cells stably expressing DCD and in vivo tumor growth in NUDE mice. Quantitative RT-PCR (***A***) confirming the expression of DCD mRNA. Y-axis indicates mean ± SD mRNA levels (normalized to HPRT) relative to controls using REST software and the expression level in SK-BR-3 pcDNA control was designated as 1. ***B,*** Xenograft growth curves of control SK-BR-pcDNA and SK-BR-3 cells stably expressing DCD in the mammary fat pads of female NUDE mice. Results are given as means ± SD of three experiments ***C***, Bioluminescence IVIS images of mice carrying luciferase-expressing cells of three representative mice shows slow tumor growth in left side implanted with SK-BR-3-pcDNA as compared with right side implanted with SK-BR-DCD cells monitored weekly with IVIS Spectrum. These cell lines were created using a Luc2 vector for expressing luciferase which produce light when in presence of D-luciferin substrate. Color scale minimum 10^3 and maximum 10^6.
